# Potential Linkage between Heavy Metal Pollution Risk Assessment and Dissolved Organic Matter Spectra in the WWTPs-River Integrated Area-Case Study from Ashi River

**DOI:** 10.3390/toxics11110904

**Published:** 2023-11-06

**Authors:** Taoyan Dai, Zhijun Li, Liquan Wang, Tienan Li, Pengpeng Qiu, Jun Wang, Haotian Song

**Affiliations:** 1 School of Water Resources and Electricity, Heilongjiang University, Harbin 150080, China; 2 Heilongjiang Province Hydraulic Research Institute, Harbin 150080, China

**Keywords:** dissolved organic matter, environmental risk assessment, human activities, parallel factor analysis, spectral index, WWTPs-river integrated area

## Abstract

Direct sewage discharge can cause severe damage to the water environment of the river. However, the impacts of dissolved organic matter (DOM) in the discharge on the original pattern of DOM and the distribution of heavy metals (HMs) in the river are little known. How to monitor such areas in a long-term and systematic manner also needs to be urgently addressed. In this paper, we characterized the DOM of the sediments in the WWTPs (wastewater treatment plants)-river integrated zone by ultraviolet-visible absorption spectroscopy (UV-vis), three-dimensional excitation-emission matrix (3D-EEM) combined with parallel factor (PARAFAC) method. The effects of WWTP on receiving waters were investigated, and the potential link between DOM and HM pollution was explored. Hg (*I_geo_*: 3.94 ± 0.65; *EF*: 44.83 ± 31.11), Cd (*I_geo_*: 1.81 ± 0.69; *EF*: 8.02 ± 2.97), Cu (*I_geo_*: 1.61 ± 0.83; *EF*: 6.85 ± 2.37), Zn (*I_geo_*: 1.55 ± 0.54; *EF*: 7.24 ± 3.58), and Ni (*I_geo_*: 1.46 ± 0.56; *EF*: 6.12 ± 1.99) in rivers were the primary risk sources of HM. The combined pollution risk indicates that the WWTPs-river integrated area is in a high pollution risk state. Moreover, *α*(254) has a significant correlation with pollution indicators and can be used as a proxy indicator. These results help to understand better the impact of WWTPs on receiving water bodies and the potential connection between DOM and HM pollution and provide new ideas for monitoring the water environment in highly polluted areas.

## 1. Introduction

In recent years, rapid population growth and industrialization have severely damaged the ecology of river systems, causing environmental pollution problems and, consequently, affecting the health of aquatic organisms and humans [1,2]. Pollutants in river systems are mainly influenced by the dissolved organic matter (DOM) content and structural characteristics [2]. Due to its complex structure and various types of attached functional groups, this affects the type and distribution of pollutants [3,4]. In addition, DOM as a mediator or controlling factor profoundly affects the distribution, transport, and final environmental fate of heavy metals (HM), organic pollutants, and nitrogen and phosphorus [5,6,7]. Therefore, the study of DOM is of great importance for protecting and managing the aquatic environment.

Sediments play an important role in river systems as the main sink and source of DOM in the overlying water [8,9]. Previous studies on sediment DOM have focused on surface sediments and have mainly concentrated on component characteristics, spatial and temporal distribution, sources, and dissolved organic carbon (DOC) cycling [10,11,12,13]. However, the link between DOM and the potential ecological risk of HM, which is a permanent pollutant and ecotoxic, has been poorly studied [14,15]. Previous studies have shown that DOM and HM can form complexes, which affect the transport transformation, ecotoxicity, and biological effectiveness of HM [16,17,18]. Among them, the humus is the primary binding ligand for HMs such as Cu [19]. The difference in the number of O-containing acidic functional groups versus N and S-containing functional groups in DOM [20], in turn, affects complexation and the behavior of HM in the environment [17,21,22]. For example, DOM can completely chelate As and reduce As mobility through the formation of covalent bonds between As(III) and organosulfur groups [23]. The correlation between DOM and HM is becoming one of the current research hotspots [7]. Some scholars have linked the potential ecological risk of HM with DOM, trying to indirectly reflect the pollution status of HM through the structural characteristics [24] and spectral indices [25] of DOM and hoping to conduct long-term automated monitoring of this. However, such studies have rarely been reported in the integrated wastewater treatment plants (WWTPs)-river zone.

As an organic compound, DOM consists mainly of humic compounds, aromatic compounds, carbohydrates, and proteins [5,26] and is the most active and sensitive part of the organic composition [27]. DOM has been extensively studied in recent years due to its ecological importance. Ultraviolet-visible absorption spectroscopy (UV-vis) and three-dimensional excitation-emission matrix (3D-EEM) fluorescence spectroscopy have been applied to characterize DOM in water bodies such as lakes, rivers, and oceans and their sediments [28,29,30]. Parallel factor (PARAFAC), the most commonly used algorithm for processing EEM data, can decompose overlapping fluorescence components [31]. Compared to fluorescence region integration (FRI) and peak picking methods, PARAFAC has the ability to quantify the analysis more accurately [32]. Compared to complex and expensive methods, such as Fourier transform ion cyclotron resonance mass spectrometry (FTICR-MS) [33], EEM provides highly valuable and comprehensive fluorescence data, such as structure and composition while being inexpensive and universally available [34,35]. Moreover, only simple sample collection, pretreatment, and non-destructive measurements are required [25]. Combined with spectroscopic techniques, DOM has the opportunity to become a long-term and accurate detection tool for developing countries. Today, several scholars have used DOM for real-time monitoring of water quality [36] and reflecting the eutrophication status of water bodies [8], thus providing early warning of water pollution. Such studies are increasingly mature [37]. However, studies on the potential link between DOM and HM pollution risk are minimal. If we try to reflect the HM pollution risk status by DOM, a lot of exploration and demonstration is needed.

The lower reaches of the Ashi River traverse Harbin, the most famous heavy industrial city in the whole of China. Compared to natural water bodies, DOM in river segments in urban areas has more complex sources and characteristics [38,39]. The functional groups, aromaticity, composition, and molecular size of DOM in urban rivers have been influenced by urbanization [40,41,42,43]. Increased heterogeneous inputs of HM, such as industrial and domestic wastewater, have led to a more complex composition and distribution of HM [44,45], especially in estuarine areas subject to complex hydrodynamic conditions. Although WWTPs remove most inorganic nutrients [46], DOM continues to flood into the river with effluent [47]. This affects the structure and composition of DOM in the river, which leads to changes in metal affinity [48]. Previous studies in such areas have primarily focused on water body black odor and eutrophication while ignoring the potential ecological risks of HM [49,50].

Therefore, this study proposes the hypothesis that the drainage of WWTPs will change the composition and properties of river sediment DOM, thus affecting the risk of HM pollution. Thus, the sampling of selected sites was carried out. UV-vis was combined with methods such as EEM-PARAFAC to study the sediments at different depths in the WWTPs-river integral area. In addition, the potential link between DOM and HM pollution risk is explored in depth by structural equation modeling (SEM). The objectives of this study were to (i) evaluate the distribution and contamination level of HM, (ii) reveal the variation of DOM components in sediments at different depths and their properties and to explore the influence of WWTPs, (iii) demonstrate the potential connection between DOM and HM contamination. This study aims to explore the effects of WWTPs on DOM and HM in receiving waters and their potential linkages, hoping to provide helpful information for long-term, automated monitoring of HM risk in such areas in developing countries worldwide. It also provides a reference for the ecological safety of dredging technology.

## 2. Materials and Methods

### 2.1. Study Area

**Figure 1:**
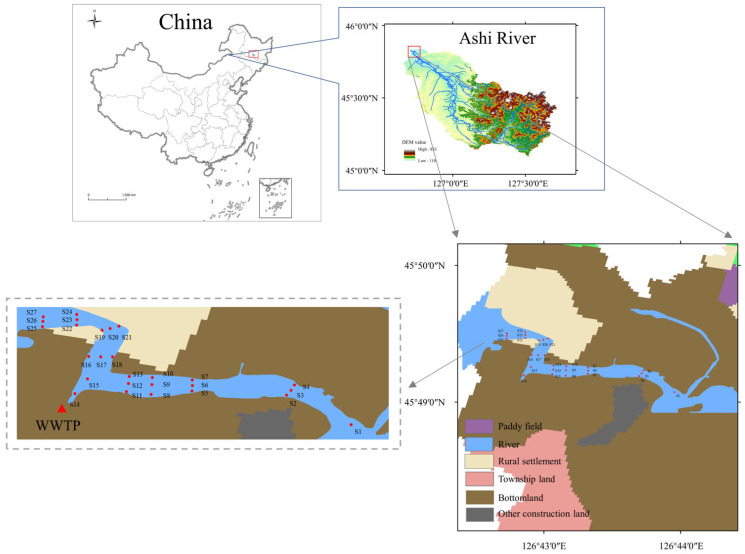
Location of the study area, sampling sites, and land use types. (DEM data from http://srtm.csi.cgiar.org/srtmdata/ accessed on 12 January 2023 and land use type data from https://www.resdc.cn/ accessed on 12 January 2023).

The Ashi River is a first-class tributary of the Songhua River (Figure 1), located in northeastern China (126°40′20″ E~127°43′33″ E, 45°5′30″ N~45°50′28″ N), with a total length of 202.8 km and a watershed area of 3493 km^2^. The upper reaches are dominated by hills and mountains, with extensive secondary forests. The lower reaches are mainly plains, primarily arable and urban land. Many factories are on both sides of the river, and much industrial wastewater is discharged into the river. Among them is a large-scale sewage treatment plant at the mouth of the river. The watershed is home to four enterprises mining zinc, lead, and copper, more than thirty enterprises mining construction materials such as marble, limestone, and granite, electroplating plants, and several factory enterprises for other purposes [51]. The developed heavy industry has caused severe damage to the ecology of the river, and the Ashi River has likewise become a major source of metal pollution in the Harbin section of the Songhua River [52].

### 2.2. Sample Collection and Preparation

Sampling has been conducted a total of three times from late September to early October 2022. Three points were taken at each section (at ¼, ½, and ¾ of the river width), and each point was sampled three times with samples from the same depth evenly mixed. The sediment samples were divided into surface (0–15 cm), middle (15–30 cm), and deep (30–50 cm) layers. Among them, two sampling sites (S14 and S15) were set at 10 m and 20 m from the outfall of the wastewater treatment plant. The estuary section was divided into I (S1–S13), II (S14–S18) and III (S19–S27). Samples were immediately returned to the laboratory in polyethylene bags, sealed. A total of 24 surface sediments (SS), 21 middle sediments (MS), and 16 bottom sediments (BS) were collected due to the actual conditions of sampling. Some sites had predominantly gravelly soils, and others had harder soils, both of which resulted in incomplete sampling. All samples were naturally air-dried, ground after removal of plant roots, and passed through a 100-mesh sieve before analysis.

The DOM was extracted by mixing sediment and Milli-Q water in a 1:5 ratio and shaken overnight at 120 r/min on a compound shaker [25]. The samples were then centrifuged at 2000 r/min for 20 min, and the supernatant was extracted and filtered through a 0.45-μm organic fiber membrane. It was also acidified with HCl to make the pH approximately equal to 3, thus preventing the potential interference of metal complexation [53], and finally stored at 4 °C in the dark for testing.

Before determining the metal content of the sediment, the 100 mL conical flask to be used was pretreated with aqua regia solution, dried, and set aside. Aqua regia is a mixture of concentrated nitric acid and concentrated hydrochloric acid with a volume ratio of 1:3. Then take 0.1 g of sample in a 100 mL conical flask, add 6 mL of aqua regia solution, put on a glass funnel, and heat the digestion on an electric heating plate. The specific practical steps were referred to the Chinese national standard HJ803-2016, and the reagents used in the experiments were of superior purity. Prior to the determination of major and trace metals, digestion was carried out under high pressure in a closed vessel with 5 mL of HNO_3_, 2 mL of HClO_4_, and 1 mL of HF. Cd, Pb, Cr, Cu, Ni, Zn, and Fe concentrations were determined by inductively coupled plasma mass spectrometry (iCAP TQ ICP-MS; Thermo Fisher, Waltham, MA, USA). Finally, the concentrations of As and Hg were determined by an atomic fluorescence photometer (AFS-230E; Haiguang Instrument, Beijing China).

### 2.3. Risk Assessment

The elemental background values in this study were obtained from a previous investigation of the background values of soils in Harbin [54]. Geochemical background concentrations were used for elements without background values available [55].

The index of geological accumulation (*I_geo_*) was proposed by Muller et al. [56]. to effectively estimate the level of HM contamination in sediments. Its equation is as follows:

(1) $$I_{geo}=\log_{2}(C_{n}/1.5B_{n})$$

*C_n_* is the actual concentration of the metal under test, and *B_n_* is the background value of the metal. The constant 1.5 is the correction factor. The enrichment factor (*EF*) method reflects the contribution of anthropogenic pollution by introducing a reference element. The common reference elements are Al, Fe, and Mn, and Fe was selected as the reference element in this study [57]. The *EF* equation is as follows:

(2) $$EF=\frac{C_{n}/C_{ref}}{B_{n}/B_{ref}}$$

*C_n_* and *C_ref_* are the concentrations of the detected elements and the reference elements, and *B_n_* and *B_ref_* are the background values of the detected and reference elements. In addition to the pollution risk assessment of individual HM, it is more important to focus on the synergistic effect of multiple HM pollution. Therefore, a variety of multi-element contamination indicators are introduced, among which the contamination factor (*C_f_*) is the fundamental indicator used for the calculation, with the following equation:

(3) $$C_{f}=C_{n}/B_{n}$$

*PLI* is the geometric mean, which assumes the same interaction between HMs. *mC_d_* is the arithmetic mean, which assumes that the effects are independent.

(4) $$PLI=\sqrt[{n}]{\prod_{i=1}^{n}C_{f}^{i}}$$

(5) $$mCd=\frac{\sum_{i=1}^{n}C_{f}^{i}}{n}$$

The Pollution Index (*PI*) contains the most polluting metal elements:

(6) $$PI=\sqrt{\frac{(C_{f}^{\mathrm{mean}}{)}^{2}+{\left(C_{f}^{\max}\right)}^{2}}{2}}$$

The potential ecological risk index (*RI*) was used to measure the ecological risk of HM in sediments by introducing a toxicity response factor (*Tr*). The *Tr* for each metal element was referred to in previous studies [58]. The *RI* was calculated as follows:

(7) $$RI=\sum_{i=1}^{n}Er^{i}=\sum_{i=1}^{n}Tr^{i}\cdot C_{f}^{i}$$

where *Er^i^* is the potential ecological risk index for individual metals. Some scholars have considered the HM background concentrations and the non-conservative behavior of sediments. In environments where the behavior of metals is dominated by material transport, the Modified Pollution Index (*mPI*) and the Modified Ecological Risk Index (*mRI*) are advocated for calculation [59,60]. The specific equations are as follows:

(8) $$mPI=\sqrt{\frac{{\left(EF^{\mathrm{mean}}\right)}^{2}+{\left(EF^{\max}\right)}^{2}}{2}}$$

(9) $$mRI=\sum_{i=1}^{n}mEr^{i}=\sum_{i=1}^{n}Tr^{i}\cdot EF^{i}$$

The thresholds of contamination levels for each index are shown in Table 1 [60,61].

### 2.4. UV–Vis Spectroscopy Analysis

The ultraviolet-visible spectra (UV-vis) of DOM were obtained by a UV-vis spectrophotometer (Evolution 220; Thermo Fisher, Waltham, MA, USA). The absorbance from 250–700 nm was obtained using Milli-Q water as a reference with a scanning interval of 1 nm. The spectral absorption coefficients were calculated using Equation (10):

(10) $$\alpha^{\prime }\left(\lambda \right)=2.303A\left(\lambda \right)/L$$

where *α*′(*λ*) denotes the uncorrected absorption coefficient for *λ*, m^−1^. *A*(*λ*) is the absorbance at *λ*. *L* is the optical range length of the cuvette, m. Tiny particles in the filtrate cause light scattering, and the absorption coefficient is usually corrected by the absorbance at 700 nm:

(11) $$\alpha \left(\lambda \right)=\alpha^{\prime }\left(\lambda \right)-\alpha \left(700\right)/700$$

*α*(*λ*) denotes the corrected absorption coefficient, m^−1^. The M value is the ratio of the absorption coefficients at 250 and 365 nm and is used to express the molecular mass (MW) of DOM. E3/E4 is the ratio of the absorbance at 300 and 400 nm and characterizes the degree of humification of DOM. E2/E4 is the ratio of absorbance at 254 to 436 nm and characterizes the relative composition of DOM autochthonous sources. *α*(254) is the absorption coefficient at a wavelength of 254 nm, which is often used to characterize the concentration of CDOM.

### 2.5. Fluorescence Spectroscopy Analysis

EEMs were acquired using a fluorescence spectrophotometer (F97 pro; Lengguang Technology, Shanghai, China) at room temperature. The instrument settings are as follows: emission wavelength (Em) range is 250–550 nm with 1 nm interval; excitation wavelength (Ex) range is 200–500 nm with 5 nm interval. The EEMs were blank-corrected by Milli-Q water [62]. The EEM standard was normalized to Raman units (R.U.) using the Raman peak measured at 350 nm [63]. The drEEMs toolbox (version 0.2.0; [64]) was used in Matlab (R2019b). The components of DOM were identified and characterized by the PARAFAC model. The model’s reliability was checked, and the number of components was determined by non-negative constraints, split-half tests, outlier checking, and residual analysis [65]. Finally, components were identified through the OpenFluor online database [66].

The fluorescence index (FI) was obtained by comparing the fluorescence intensity of Em at 470 and 520 nm at Ex = 370 nm [67]. The biological index (BIX) was obtained by comparing the fluorescence intensity of Em at 380 nm with that at 430 nm at Ex = 310 nm [68,69]. The humification index (HIX) was calculated from Ex at 254 nm, the ratio of Em (435–480 nm) to Em (300–345 nm) spectral area [70,71]. At Ex = 310 nm, the freshness index (β:*α*) was obtained by comparing the fluorescence intensity of Em at 380 nm with the maximum fluorescence intensity of Em in the 420–435 nm interval [68,69].

### 2.6. Statistical Analysis

The data were tested for reliability using IBM SPSS Statistics 25 then structural equation modeling (SEM) was constructed using IBM SPSS AMOS 26. Correlation analysis, regression scores, and principal component analysis (PCA) between variables were performed using SPSS 25. Redundancy analysis (RDA) was performed through Canoco 5. Sampling point location maps, as well as kriging interpolation for heavy metals, were plotted through ArcGIS 10.2. The remaining graphs were plotted using Origin 2021.

## 3. Results and Discussion

### 3.1. Heterogeneity of Elements in the WWTPs-River Integrated Area

The WWTPs-river integrated area has significant HM loadings, which are influenced by the interaction between natural conditions and anthropogenic activities (Table 2). The morphology of the surface water is one of the factors affecting HM pollution. Vidy Bay of Lake Geneva is severely threatened by heavy metals [72]. The maximum content of Cd, Pb, and Zn in Vidy Bay sediments was 454.26%, 573.4%, and 174.83% of that in Ashi River. Even if the climatic and geochemical conditions of the lake are similar to the river, the chemical composition is significantly different, and the distribution of heavy metals in the sediments is different. Rivers have a diverse and variable water chemical composition, while lakes have slow water exchange and poor hydrodynamic conditions, which can easily cause the deposition of particulate matter. Moreover, particulate matter has strong adsorption of heavy metals. Compared to Vidy Bay and Ashi River, Lake Mariout has a strong self-purification capacity as a wetland [73]. Furthermore, artificial wetlands are an essential way to manage urban wastewater. Different plants are selected for the targeted treatment depending on the excess elements. For example, *Ludwigia* stolonifera has considerable potential for metal recovery in Cu, Fe, and Mn, with removal rates of 86%, 74%, and 93%, respectively [74]. In contrast, *Vetiveria zizanioides* was more effective in the removal of Zn, Fe, Cu, Cd, and Pb [75]. In addition to such mild treatment measures, dredging of the riverbed, creation of channels around the estuary to prevent effluent discharge, and closure of existing plants in the Golden Horn area improved the recovery of heavy metal levels in the bottom sediment [76]. The above methods can be used to efficiently manage HM pollution in the Ashi River. Biebrza River is naturally favored and less influenced by anthropogenic activities. The heavy metal load in the WWTPs-river integrated area within the basin is much less than in the rest of the area [77]. The ground-rotation bias forces also affect the enrichment of metal elements (Figures S1–S3). Sediments tend to accumulate on the convex shore, where the flow velocity is slow and water exchange is slow. HMs in SS, MS, and BS are enriched on this side, so more attention should be paid to HM pollution on the convex shore when dredging projects are carried out.

**Figure 2:**
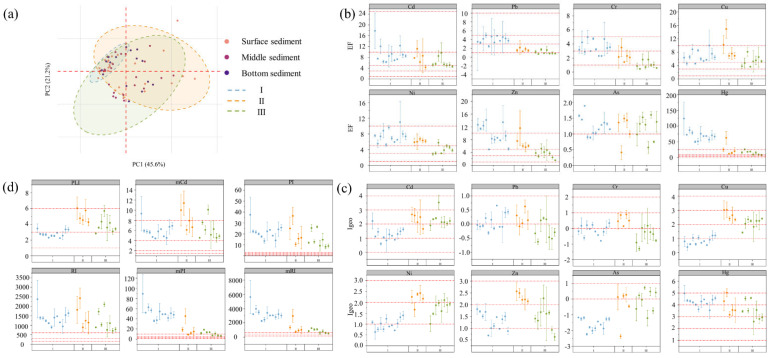
Along-river variation of heavy and pollution risk in WWTPs-river integrated area. (**a**) results of principal component analysis; (**b**) enrichment factor (*EF*); (**c**) ground accumulation index (Igeo); (**d**) integrated pollution index.

The PCA results (Figure 2a) describe the elemental behavior under the influence of WWTPs. 45.6% versus 21.2% of the variance is explained by PC1 (Ni, 0.916; As, 0.862; Cu, 0.829; Cd, 0.811; Zn,0.581) and PC2 (Cd, 0.809; Pb, 0.746; Zn, 0.643). The spatial distribution of heavy sediment metals was heavily influenced by WWTPs, with significant and intensive enrichment in river section I. Ni, As, Cu, Cd, and Zn, which constitute PC1, produced large fluctuations in both river section II and river section III. Moreover, Ni, As, Cu, and Cd exhibit a better correlation with Fe (Figure S4a), indicating that the differences in clay mineral-related elements such as Fe dominate the heterogeneity of HM. The HM content of sediments is influenced by particle size. Muddy sediments composed of aluminosilicate minerals (i.e., clay minerals) have a high cation exchange capacity and specific surface area. Therefore, it is easier to adsorb metal elements. However, sandy sediments composed of carbonates and quartz have a lower cation exchange capacity and specific surface area and are less prone to adsorb metal elements [78]. Except for Hg, the HM concentrations of sediments at different depths exhibit similar trends (Table S1). The surface sediments were vulnerable to human activities, and the concentration of HM showed a significant fluctuation. The potential link between HM and DOM will be analyzed in detail in subsequent sections.

**Table 2:** Comparison of heavy metal content in sediments from the Ashi River WWTPs-River Complex with other direct sewage discharge areas around the globe.

Location	Source	Cd	Pb	Cr	Cu	Ni	Zn	As	Hg	Fe
Ashi River, Harbin	This Study	0.19–2.23	17.45–86.85	32.00–254.49	33.38–384.71	42.05–224.87	131.01–894.57	1.87–19.85	0.38–8.98	4.98–62.56
Vidy Bay, Lausanne, Switzerland	[72]	0.74–10.13	43–498	-	75–362	-	170–1564	8–22	0.24–8.98	18.2–47.8
Biebrza River, Poland	[77]	-	-	8.4–28.3	3.1–54.7	-	17.4–162.7	-	-	1.07–1.57
Cape Town, South Africa	[79]	0.25–1.77	-	-	-	-	178.16–949.87	5.70–34.66	0.01–0.3	-
Lake Mariout, Egypt	[73]	0.9–1.0	91.6–93.0	79.8–89.9	129.4–148.5	46.3–59.8	-	-	-	-
Golden Horn, Istanbul, Turkey	[76]	0.1–2	8–50	26–110	70–135	10–38	70–260	-	-	-

Note: Cd, Pb, Cr, Cu, Ni, Zn, As, Hg in mg kg^−1^ and Fe in g kg^−1^.

### 3.2. Risk of Heavy Metal Pollution

#### 3.2.1. Mono-Metal Pollution Risk

According to *I_geo_*, the contamination levels of metals were as follows: Hg (3.88 ± 0.84, Severely) > Cd (1.85 ± 0.81, Moderately) > Cu (1.72 ± 0.91, Moderately) > Zn (1.58 ± 0.66, Moderately) > Ni (1.54 ± 0.60, Moderately) > Pb (0.02 ± 0.45, Slightly) > Cr (−0.02 ± 0.66, Unpolluted) > As (−0.85 ± 1.00, Unpolluted). According to *EF*, the contamination levels of metals were as follows: Hg (42.12 ± 33.08, Extremely) > Cd (7.94 ± 3.88, Moderately-severely) > Zn (7.16 ± 4.22, Moderately-severely) > Cu (7.14 ± 3.05, Moderately-severely) > Ni (6.24 ± 2.46, Moderately-severely) > Cr (2.53 ± 1.63, Minorly) > Pb (2.51 ± 1.54, Minorly) > As (1.19 ± 0.39, Minorly). Similar results emerged from both methods, where Hg, Cd, Cu, Zn, and Ni were the primary contaminants. There is a strong correlation between Cd, Cu, Zn, and Ni (Figure S4a), indicating that these HMs have similar sources or produce compound pollution. Cd usually originates from the use of fertilizers and pesticides in agricultural activities and may also originate from the plastics industry along with Zn [80]. Cu and Zn are associated with industrial activities, such as mining, smelting, and metal processing in the watershed. As a pro-copper element, Zn exhibits similar properties in the water column. It can combine with S^2−^ produced by organic matter in sediment under anaerobic conditions to form sulfide with very small solubility and thus precipitate. Ni and Cu are also present in the wastewater of electroplating and semiconductor industries.

Hg may originate from the combustion of fossil fuel coal or petroleum products. The correlation between Hg and other elements is low, and its pollution source may also be influenced by its own physicochemical properties, sediment content, and organic matter content in the sediment. As, Cr, and Pb contamination is low and little affected by anthropogenic inputs. All metal elements produced more significant fluctuations before and after WWTP (Figure 2b,c). It indicates that DOM changes the environmental behavior of HM. Under uncontaminated conditions, most of the heavy metals are distributed in the mineral lattice and present in Fe and Mn oxides that act as the coating film of the particulate matter. The anthropogenic heavy metals are mainly present on the surface of the particulate matter in the form of adsorption or combined with organic matter in the particulate matter.

#### 3.2.2. Integrated Pollution Risk

The comprehensive pollution evaluation of HM is shown in Figure 2d. Each evaluation index shows significant fluctuations before and after WWTP. The results of *PLI*, *mC_d_*, *PI*, and *RI* were greatest in river section II, while the results of *mPI* and *mRI* were greatest in river section I. The reason for the contrasting trends is that *mPI* and *mRI* were calculated using *EF* for interpreting the petrogenesis and depositional input of metal elements [60]. In some regions where Fe, Al, and Mn are less affected by human activities, the introduction of *EF* enhances the robustness of *mPI* and *mRI* to metal contamination [25,57]. However, it is less applicable in the WWTPs-river integrated zone. Fe, as a reference element, showed large fluctuations in the region. In addition to Fe enrichment due to sediment transport, the anthropogenic input is responsible for the sudden changes in Fe content. The values of most of the indicators exceed the maximum threshold for classification, which is explained by the fact that Hg is the element that contributes the most to pollution in the region, with concentrations well above the soil background values. Again, Hg has the highest toxicity response factor. *PLI* provides a more detailed interpretation relative to *mC_d_* (Figure S5a). *PI* and *RI* provide a more robust assessment relative to *mPI* and *mRI* because they are not influenced by the anthropogenic input of Fe (Figure S5b,c). Also, *RI* takes into account the differences in toxicity between HM.

Both single metal evaluation and comprehensive pollution evaluation show that the WWTPs-river integrated area is the hardest hit area for HM pollution, and the environmental behavior of HM is affected in this area. Therefore, long-term regulation and early warning are carried out for this area to meet the strict environmental requirements of contemporary society. As, Cd, and Cr are carcinogenic risks, Hg can be highly harmful to the human respiratory and central nervous systems [81]. Therefore, we advocate the use of such indicators for quantitative analysis rather than just for classification. Among the above indicators, we selected *PLI*, *RI*, and *PI* as evaluation indicators for such regions.

### 3.3. Component and Spectral Indices of DOM

**Figure 3:**
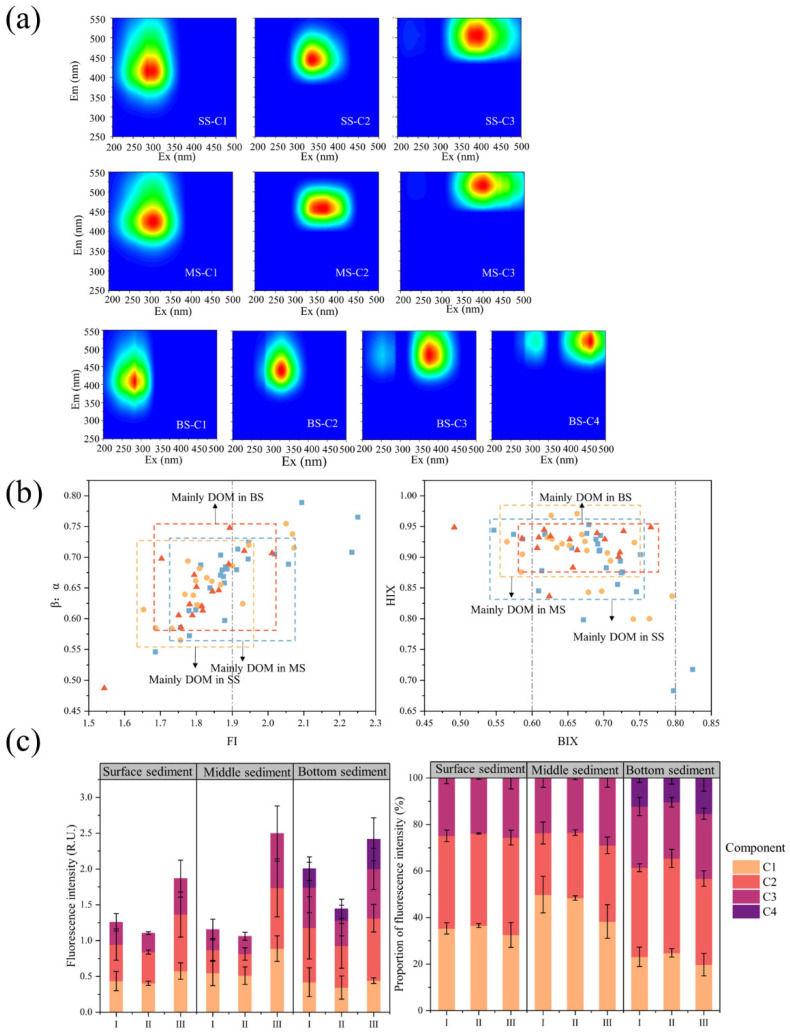
DOM fractions and spectral indices. The subplots show (**a**) the surface, middle, and bottom sediment DOM fractions, (**b**) the spectral index of the sediment, (**c**) the variation of DOM fractions in different river sections.

SS-C1 (Ex/Em = 320/415), MS-C1 (Ex/Em = 340/425), and BS-C1 (Ex/Em = 320/407) are similar to humic peaks C and M (Figure 3a). Liu et al. [82] suggest that C1 is humus produced by human activities. However, Dainard et al. [83] suggest that C1 production originates from microbial reprocessing or phytoplankton degradation. With the corroboration of spectral indices, the present study prefers the latter view. SS-C2 (Ex/Em = 365/445), MS-C2 (Ex/Em = 390/458), and BS-C2 (Ex/Em = 360/441) belong to the humic-like peak C, reported in recycled water effluent DOM and considered to be associated with wastewater discharge [25]. SS-C3 (Ex/Em = 275,415/506) and MS-C3 (Ex/Em = 280, 415/515) are similar to soil fulvic acid and are of native microbial origin. This fraction is of microbial origin but is removed in visible light due to photodegradation. It is difficult to obtain in natural waters because of its very rapid production and disappearance [84,85]. The production of this component may be related to the reaction of primary optical processes and photo-flocculation products [86]. However, the interaction between biodegradation and photodegradation is difficult to predict. Photodegradation might promote biodegradation by converting high molecular-weight substances to low molecular-weight substances such as aldehydes and acids. Photodegradation may also have an inhibitory effect on biodegradation [87]. The components in the surface sediments are essentially similar to those in the middle sediments, but the wavelengths of the components in the middle sediments are significantly redshifted. BS-C3 (Ex/Em = 295, 395/483) is similar to soil fulvic acid, and its production is due to selective loss and preservation of DOM under prolonged dark conditions [88]. BS-C4 (Ex/Em = 345, 465/521) is similar to soil fulvic acid peak E. No studies related to this fraction were found in OpenFluor. The present study speculates that the formation of this component may be similar to BS-C3. Information on the origin of this fraction and the related properties should be included in subsequent research work.

The FI values of DOM (1.86 ± 0.13, 1.54–2.25) indicate that DOM is weakly aromatic and mainly of microbial origin [89]. Large fluctuations in sediment FI due to inputs from land-based sources at some points (Figure 3b). HIX (0.90 ± 0.05, 0.68–0.97) reflects a low degree of DOM humification. DOM is mainly derived from organisms and aquatic bacteria, and this result corroborates with FI [90]. However, the BIX (0.67 ± 0.06, 0.49–0.82) and β:*α* (0.66 ± 0.06, 0.49–0.79) indices reflect a moderate level of bioactivity with simultaneous influence by endogenous and exogenous sources. This seems to be at variance with the overwhelming endogenous input shown by the FI and HIX indices, contrary to other relevant studies. This may be because endogenous DOM produced by microorganisms and algae accounts for a portion of the DOM, and another portion is not derived from unusual terrestrial humic substances but from the drainage of non-endogenous WWTPs. The drainage input of DOM resulted in a reduced share of endogenous DOM while possessing a lower degree of humification. Secondly, indirect photodegradation is inhibited when the dissolved oxygen concentration is reduced, thus failing to enhance the degree of humification [91]. The degree of humification, aromaticity, and molecular weight of DOM gradually increased with increasing depth, in contrast to the conclusion reached by Li et al. [92]. The reason for this is the small percentage of anaerobic microbial communities in the sediments of this study area. Thus, the microbial activity gradually decreases with increasing depth. Humic substances fail to be reused by biological communities, and similarly, high molecular weight DOM is not converted into smaller fragments by microbial uptake [93]. Aromatic compounds are preferentially preserved during sediment accumulation.

The molecular weight of DOM is smaller at the WWTPs-affected section II (Table S2). WWTPs bring large amounts of organic matter to rivers. The organic matter is broken down by microorganisms into simple compounds, and these materials then form monomer molecules of humus as they continue to be acted upon by microorganisms. Humus-like substances are similarly derived from the decomposition of aquatic plants and algae due to the high level of eutrophication and rapid algal blooms in the area [94]. The sudden increase in *α*(280) of sediment DOM in section II (Table S2) indicates that the outflow of WWTPs leads to an elevated sediment DOM-like protein content. At low temperatures, microorganisms break down proteins and store DOM to maintain basic functions, and humus is released as a breakdown product [95]. In addition, protein-like adsorption on minerals would contribute to forming certain fractions of humic substances [96]. The sediments are then influenced by hydrodynamic conditions to move further downstream. In river section III, the Ashi River is about to join the Songhua River, creating more static hydrodynamic conditions at this location where sediments settle in large quantities.

Under these conditions, microbial degradation becomes stronger and contributes to the formation of humus. At the same time, humus is highly biostable and hydrophobic and is not easily transformed by microbial uptake in aqueous environmental systems [97]. Therefore, the fluorescence intensity of each component of sediment DOM reaches its maximum at river section III (Figure 3c).

### 3.4. Potential Connection between DOM and HM

**Figure 4:**
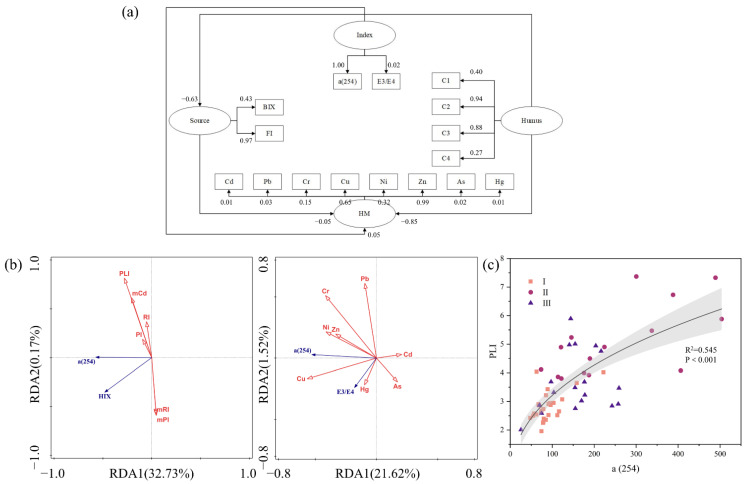
Potential association of DOM with HM. (**a**) SEM model, (**b**) RDA analysis, (**c**) linear regression of *PLI* with *α*(254).

Sixteen observed variables (C1, C2, C3, C4, Cd, Pb, Cr, Cu, Ni, Zn, As, Hg, BIX, FI, *α*(254), E3/E4) were selected and divided into four potential variables (Humus, HM, Index, Source) for SEM model construction (Figure 4a). Reliability and validity tests were performed on the data to ensure that the SEM model was constructed smoothly and with reliability, and the specific parameters are shown in Table S4. The regression path coefficient between “Humus” and “HM” was −0.85, indicating that humic substances play a more significant role in the migration of HM. The factor loadings of Zn and Cu were 0.99 and 0.65, respectively, are more affected by DOM than other metallic elements. The factor loading of C2 was 0.94, indicating that the discharge of DOM in the wastewater had a severe impact on the distribution and morphology of the original HM in the WWTPs-river integrated zone. The loading factor of C2 was 0.94. The factor loading of C3 was 0.88, which has a higher molecular weight than soil fulvic acid and also possesses a stronger binding potential.

DOM has a vital role in regulating the morphology and distribution of metal elements. With increasing depth, the concentration of Cr in BS was much greater than in SS and MS (Table S3). Again, the fluorescence intensity of each component reached its maximum in BS. With increasing DOM, the bioreduction of Cr in the soil was stronger than the chemical reduction, thus enhancing the adsorption of Cr to the soil [98]. The concentration of Zn and Cd decreases with the increase in depth. The inhibitory effect is more obvious when the pH is higher and the metal solubility is lower. Among them, the inhibition of Zn was greatest when the pH was 7–7.5 [99]. This phenomenon was also found in the present study, where a total of 19 samples with pH between 7 and 7.5 were found to have a concentration of Zn of 235.38 ± 66.66 mg kg^−1^ and 44 samples with pH not in this range. The concentration of Zn was found to be 402.73 ± 184.38 mg kg^−1^. It has also been suggested that the decrease in Zn in sediments is due to the adsorption of Fe/Mn oxides and that the oxidative dissolution of Fe/Mn oxides affects the release of Zn [100]. The sorption of Cd and Pb increases with increasing concentrations of metal elements. The increase in DOM inhibits the sorption of Cd by the soil. The trend of Pb is more complex. Zhu et al. [101] concluded that when the Pb concentration reaches 150 mg L^−1^, its trend with DOM will be the same as that of Cd. When the concentration is small, DOM does not have a significant effect on Pb. This is because when the concentration reaches a certain level, DOM occupies more sorption sites as the DOM content increases. Fe has a stronger affinity with Cu than Zn. The concentration of Cu in river section II and river section III was 3.84 and 2.55 times higher than that in river section I, respectively. The increase of each component with *α*(280) in river sections II and III indicates that the humic-like and protein-like contents were elevated. Furthermore, the affinity of humus-like and protein-like to Cu was stronger. Compared to the low molecular weight humic-like fractions, the high molecular weight counterparts have more substantial binding potential. Thus, C3 and C4 have higher metal binding potentials. It has also been suggested that protein-like fractions have higher binding potentials to metals compared to humic-like fractions [102]. Humic-type DOM is an essential ligand for complexation with Fe. The complex of DOM with Fe has better stability and effectively reduces biodegradation. In addition to anthropogenic input and natural accumulation, this is one of the reasons for Fe enrichment. In addition, the concentration of Cu ions is also one of the factors affecting Fe. The coexistence of ions on mineral surfaces leads to competitive adsorption, which promotes the dissolution and release of Fe. The concentration of As is usually controlled by adsorption on metal oxide surfaces, especially Fe(oxy)(hydr)oxides. In the present study, Fe was also extremely correlated with AS (Figure S4a, R = 0.87, *p* < 0.001). Under oxidizing conditions, Fe(oxy)(hydr)oxides are adsorbed via arsenate, which reduces the mobility of As; thus, As may be enriched in Fe-rich soils or sediments through the cumulative adsorption of Fe(oxy)(hydr)oxides [103]. DOM has some inhibitory effect on Hg. The Hg concentration tended to decrease with increasing sediment depth. Similarly, in river section II and river section III, the concentration of Hg decreased abruptly. DOM affects the redox reaction of Hg and anaerobic microorganisms. It has been suggested that DOM enhances the methylation of Hg, especially under sulfated conditions [104]. The resulting methyl-containing compounds can be more toxic than the corresponding inorganic substances and are more harmful to humans and organisms.

*α*(254) is a more stable parameter for pollution risk and individual HM concentrations (Figure 4b). *PLI* can adequately express the combined ecological risk of HM with an excellent fit to *α*(254) (Figure 4c). Our results support that *α*(254) is a potential parameter that can be applied in long-term, automated monitoring of HM and provide early warning of HM pollution. The inclusion of DOM in the monitoring allows for exploring the ecological processes and environmental effects of the WWTPs-river integrated zone through the dynamic processes of the DOM pool. Similarly, spectral indices provide a better reflection of water pollution and eutrophication levels in the overlying water (unpublished data for this region). In addition, *α*(254) can be equally applied to the remote sensing of water bodies [105] and to the monitoring of the removal of certain organic compounds during water treatment [106].

## 4. Conclusions

The distribution pattern of heavy metal in the WWTPs-river integrated area of the Ashi River was studied. The primary pollutants are Hg, Cd, Cu, Zn, and Ni. The combined pollution level of heavy metal in this area is high, and more stringent regulatory measures need to be implemented. *PLI*, *PI*, and *RI* are more suitable indicators for the comprehensive evaluation of WWTPs-river integrated area. Moreover, *α*(254) can be used as a substitute indicator for long-term monitoring of heavy metal pollution risk. In conclusion, *α*(254) is a highly promising parameter with broad citation prospects. Of course, the idea must be justified in other regions due to the different amounts and types of DOM discharged from WWTPs.

The composition, source, and structure of DOM in the region were studied using EEM-PARAFAC, and humic-like substances Component 1 and Component 2 were resolved with soil fulvic acid Component 3 and Component 4. There is a significant spatial variation in the optical characteristics of DOM, indicating that WWTPs have a severe impact on receiving rivers. The WWTPs drainage contains a large amount of DOM, which affects the original pattern of river DOM. In subsequent work, we will study more WWTP-river integration zones to verify the role of *α*(254). We will also use FT-ICR MS to study the relationship between DOM and heavy metals at the molecular level.

## Figures and Tables

**Table 1:** The thresholds for classification of pollution status.

Mono-Metal Pollution Risk				Integrated Pollution Risk					
*I_geo_*	Qualification	*EF*	Enrichment	*mC_d_*	*PI*	*mPI*	Qualification	*RI*/*mRI*	Risk
<0	Unpolluted	1	No	<1.5	<0.7	<1	Unpolluted	<150	Low
0–1	Slightly	1–3	Minorly	1.5–2	0.7–1	1–2	Slightly	150–300	Moderate
1–2	Moderately	3–5	Moderately	2–4	1–2	2–3	Moderately	300–600	Considerable
2–3	Moderately-severely	5–10	Moderately-severely	4–8		3–5	Moderately-heavily	>600	Very high
3–4	Severely	10–25	Severely	8–16	2–3	5–10	Severely		
4–5	Severely-extremely	>25	Extremely	16–32	>3	>10	Heavily		
>5	Extremely			32			Extremely		

## Data Availability

The data presented in this study are available on request from the corresponding author. The data are not publicly available due to requirements of the research institution.

## Supplementary Materials

The following supporting information can be downloaded at: https://www.mdpi.com/article/10.3390/toxics11110904/s1, Figure S1: Distribution of trace metals in surface sediments under kriging interpolation; Figure S2: Distribution of trace metals in the middle sediment under kriging interpolation; Figure S3: Distribution of trace metals in the bottom sediment under kriging interpolation; Figure S4: Correlation analysis between DOM spectral index and (a) actual concentration of trace metals, (b) *I_geo_* index for each trace metal, (c) *EF* index for each trace metal, and (d) combined contamination risk; Figure S5: Comparison of different risk assessment methods. (a) *mC_d_* v.s. *PLI*, (b) *mPI* v.s. *PI*, (c) *mRI* v.s. *RI*; Table S1: Vertical distribution of heavy metals in each river section; Table S2: Exponential variation trend of DOM spectrum; Table S3: Changes of heavy metal content at different depths; Table S4: SEM model index and its requirements.
