# Supplementary material for: Potential Linkage between Heavy Metal Pollution Risk Assessment and Dissolved Organic Matter Spectra in the WWTPs-River Integrated Area-Case Study from Ashi River
Source: Toxics. 2023 Nov 6;11(11):904. doi: 10.3390/toxics11110904 (PMC10674235; doi:10.3390/toxics11110904)
Supplement: Supplementary file 1 [file toxics-11-00904-s001.zip › toxics-2693186-supplementary.pdf]

SupplementaryMaterials

Potential Linkage between Heavy Metal Pollution Risk Assessment and Dissolved Organic Matter Spectra in the WWTPs-River Integrated Area-Case Study from Ashi River

Taoyan Dai, Zhijun Li, Liquan Wang, Tienan Li, Pengpeng Qiu, Jun Wang and Haotian Song

Table S1. Vertical distribution of heavy metals in each river section.

Depth (cm)		Cd	Pb	Cr	Cu	Ni	Zn	As	Hg	Fe
	I	0.29±0.09	38.10±8.74	78.91±19.66	54.24±11.48	69.07±13.71	272.48±70.30	3.66±0.93	3.53±1.10	12.53±2.78
0-15	II	1.12±0.69	42.63±12.42	142.49±42.52	250.71±77.92	171.49±35.71	634.30±170.36	12.07±6.43	3.64±2.14	43.59±9.20
	III	0.85±0.53	36.93±19.95	88.45±66.80	157.41±55.54	122.23±39.21	399.37±252.94	10.13±5.34	2.62±1.21	42.23±1.04
	I	0.42±0.19	37.13±7.85	93.06±27.01	55.61±12.84	70.10±16.04	298.79±77.21	3.73±1.07	3.67±1.90	13.17±2.36
15-30	II	0.65±0.21	40.66±10.89	121.04±34.72	240.99±89.11	172.49±31.98	505.40±112.28	10.47±5.11	3.49±2.31	35.98±6.61
	III	0.65±0.19	28.96±11.47	65.29±42.22	121.39±33.10	109.76±17.10	286.95±106.72	12.13±3.60	2.11±1.26	40.05±9.47
	I	0.33±0.09	46.80±8.19	99.33±21.07	63.88±17.04	78.03±12.20	224.70±54.89	3.66±0.87	3.53±1.04	13.15±2.10
30-50	II	0.61±0.30	46.40±9.89	146.40±47.47	138.66±18.84	135.13±34.43	499.88±69.74	7.65±3.98	2.88±1.49	28.66±9.14
	III	0.62±0.11	36.25±3.87	82.64±25.61	151.50±63.43	138.68±42.13	246.95±95.83	11.93±5.75	1.79±0.85	37.86±5.13
	I	0.34±0.12	39.60±7.92	84.25±19.07	54.67±10.17	69.01±11.56	272.34±64.65	3.77±0.86	3.64±1.01	12.96±1.94
0-50	II	0.79±0.22	43.23±7.70	136.64±24.78	210.12±49.38	159.70±26.94	546.53±68.53	10.06±4.51	3.34±1.58	36.08±3.75
	III	0.75±0.35	33.48±8.40	75.37±29.14	139.18±24.21	118.72±23.66	326.29±117.32	11.54±4.36	2.39±1.15	40.94±8.51

Note: The unit of Fe is g kg^{-1}, and the unit of all other metallic elements is mg kg^{-1}.

Table S2. Exponential variation trend of DOM spectrum.

	Longitudinal			Transverse		
	SS	MS	BS	I	II	III
FI	1.91±0.13	1.84±0.11	1.81±0.10	1.84±0.11	1.86±0.06	1.89±0.18
β：α	0.67±0.06	0.65±0.05	0.64±0.06	0.66±0.06	0.67±0.03	0.65±0.18
BIX	0.68±0.07	0.67±0.06	0.65±0.07	0.67±0.07	0.68±0.03	0.66±0.08
HIX	0.88±0.07	0.90±0.05	0.92±0.03	0.90±0.05	0.92±0.03	0.89±0.07
a(280)	105.44±71.76	125.03±87.83	113.13±71.94	68.40±27.45	195.52±98.92	117.69±50.92
a(254)	143.47±101.83	156.49±111.22	148.06±92.98	89.15±34.36	251.87±137.25	156.91±63.72
M	5.32±1.63	4.93±1.49	4.81±1.30	4.38±0.76	6.31±1.47	5.05±1.76
C1	0.47±0.13	0.64±0.23	0.40±0.16	0.47±0.17	0.42±0.14	0.64±0.22
C2	0.58±0.27	0.47±0.34	0.74±0.36	0.50±0.31	0.44±0.22	0.83±0.32
C3	0.36±0.19	0.41±0.32	0.54±0.32	0.36±0.23	0.29±0.14	0.64±0.33
C4	-	-	0.28±0.23	0.26±0.17	0.16±0.13	0.41±0.30

Table S3. Changes of heavy metal content at different depths.

Depth (cm)	Cd	Pb	Cr	Cu	Ni	Zn	As	Hg	Fe
0-15	0.64±0.55	38.63±14.11	94.68±50.63	126.55±90.35	106.56±49.33	385.45±217.31	7.41±5.59	3.26±1.47	28.25±16.84
15-30	0.55±0.23	35.06±10.95	89.48±40.31	118.79±84.63	106.16±44.30	339.59±130.23	8.12±5.08	3.09±1.95	27.48±14.24
30-50	0.51±0.23	43.38±9.14	108.83±42.01	114.63±55.21	114.82±42.37	317.65±143.87	7.49±5.22	2.78±1.36	25.72±12.00

Note: The unit of Fe is g kg^{-1}, and the unit of all other metallic elements is mg kg^{-1}.

Table S4. SEM model index and its requirements.

Index	Index name	requirement	This article
CMIN/DF	Cardinality/Freedom	＜5 good，＜3 excellent	1.280
RMR	Root mean square residuals	＜0.08 good，＜0.05 excellent	104.240
RMSEA	Approximate root mean square residuals	＜0.08 good，＜0.05 excellent	0.068
NFI	Normative fit index	＞0.8 good，＞0.9 excellent	0.860

CFI	Comparative fit index	＞0.8 good，＞0.9 excellent	0.964
IFI	Incremental fitting index	＞0.8 good，＞0.9 excellent	0.966
GFI	Goodness-of-fit index	＞0.8 good，＞0.9 excellent	0.826
AGFI	Adjusted goodness-of-fit index	＞0.8 good，＞0.9 excellent	0.722

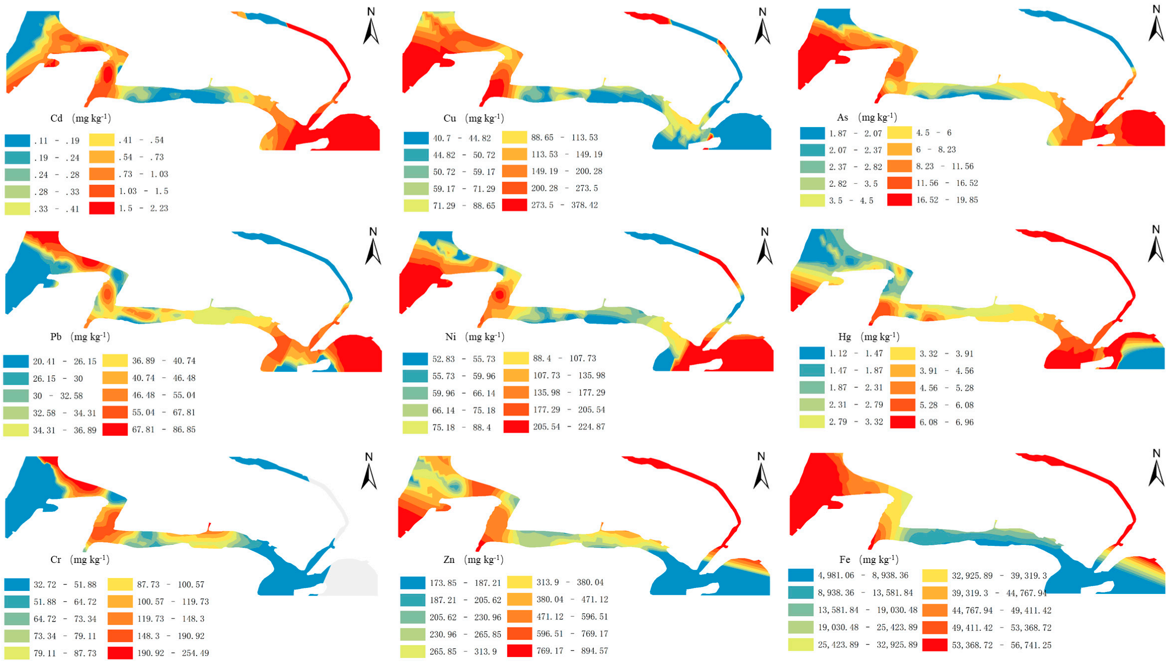

Figure S1. Distribution of trace metals in surface sediments under kriging interpolation.

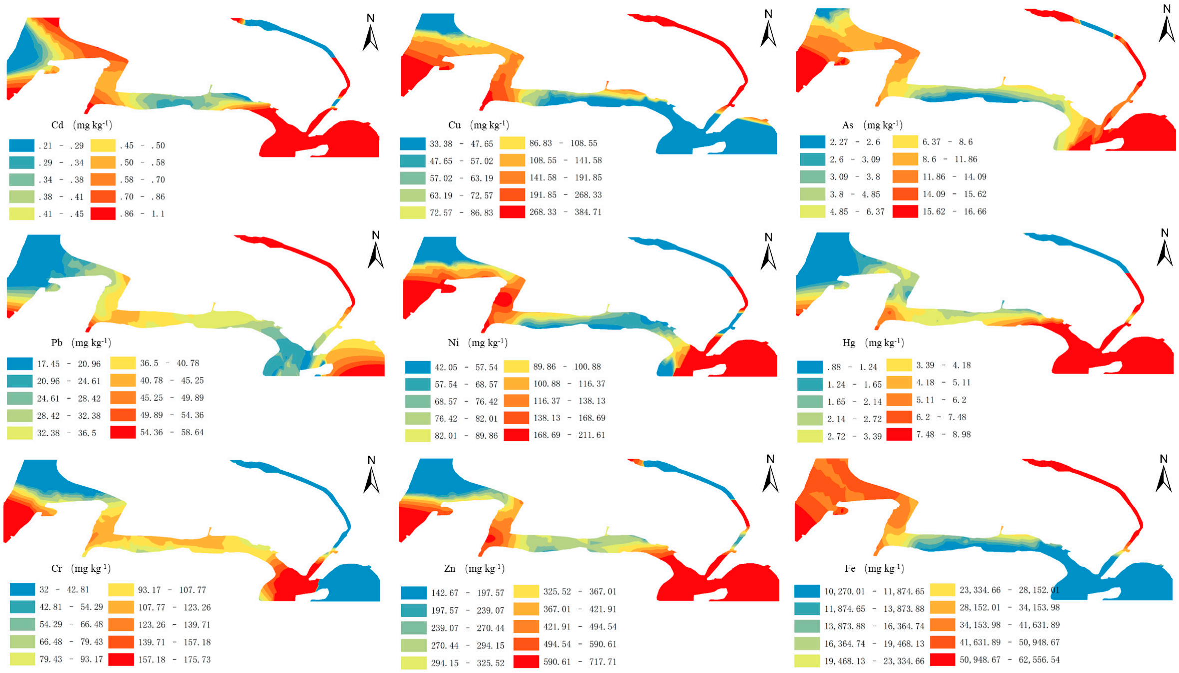

Figure S2. Distribution of trace metals in the middle sediment under kriging interpolation.

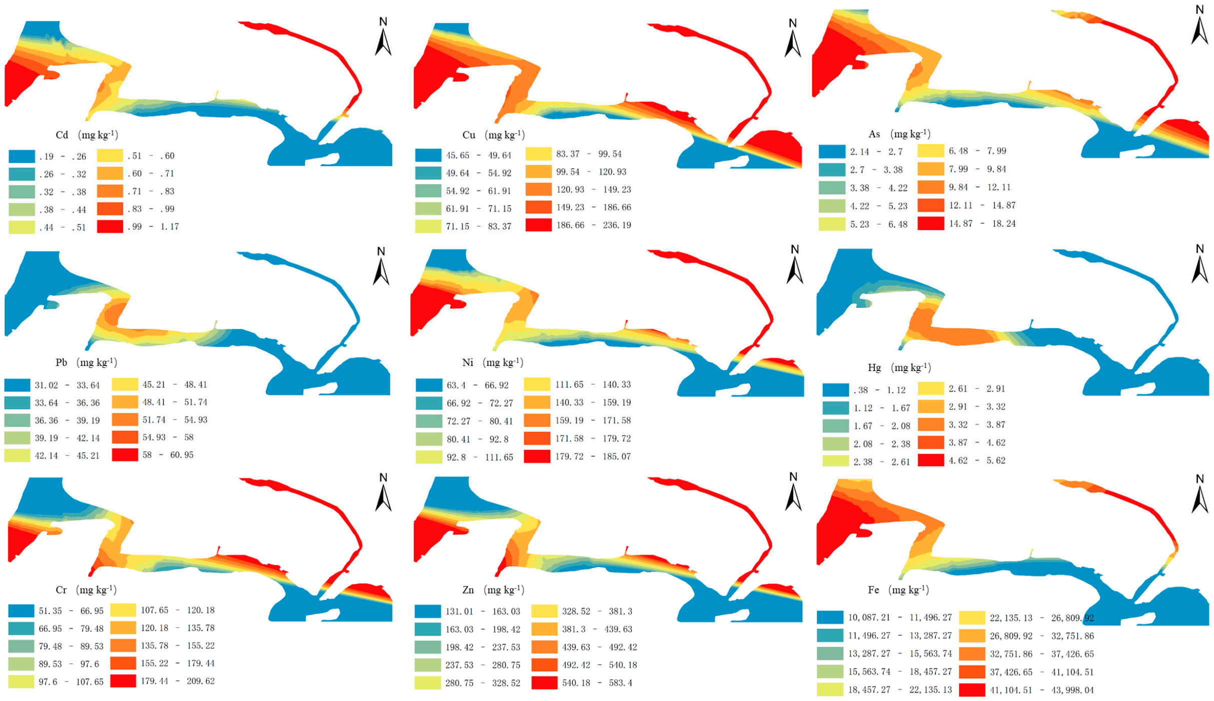

Figure S3. Distribution of trace metals in the bottom sediment under kriging interpolation.

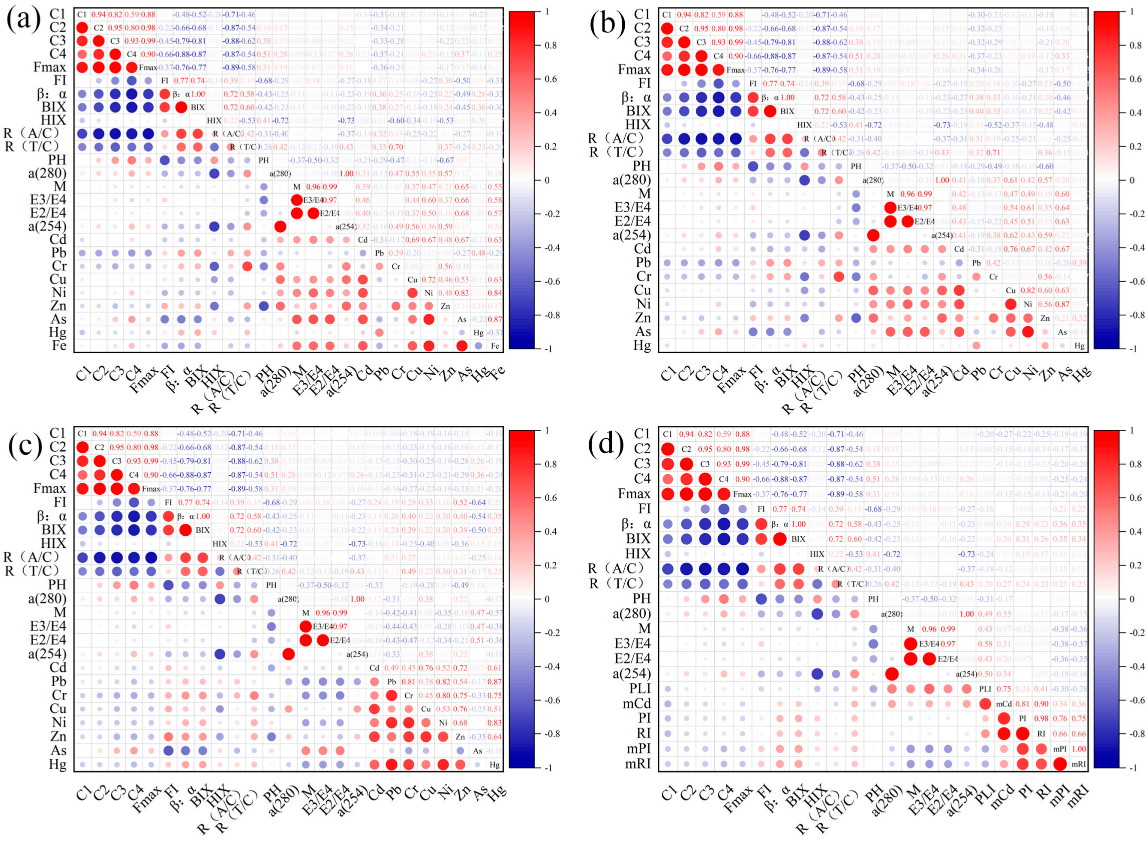

Figure S4. Correlation analysis between DOM spectral index and (a) actual concentration of trace metals, (b) Igeo index for each trace metal, (c) EF index for each trace metal, and (d) combined contamination risk.

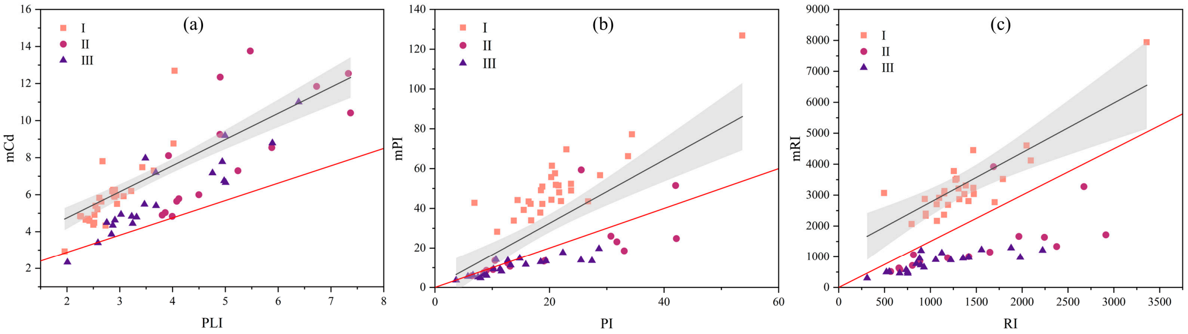

Figure S5. Comparison of different risk assessment methods. (a) mC_d v.s. PLI, (b) mPI v.s. PI, (c) mRI v.s. RI.
